# DkmiR397 Regulates Proanthocyanidin Biosynthesis via Negative Modulating *DkLAC2* in Chinese PCNA Persimmon

**DOI:** 10.3390/ijms23063200

**Published:** 2022-03-16

**Authors:** Fatima Zaman, Meng Zhang, Ying Liu, Zhilin Wang, Liqing Xu, Dayong Guo, Zhengrong Luo, Qinglin Zhang

**Affiliations:** Key Laboratory of Horticultural Plant Biology, Ministry of Education, Huazhong Agricultural University, Wuhan 430070, China; fatimazaman@webmail.hzau.edu.cn (F.Z.); zhangmeng2015430@163.com (M.Z.); liuying1991@webmail.hzau.edu.cn (Y.L.); wzl17313294782@163.com (Z.W.); liqingxu@mail.hzau.edu.cn (L.X.); guoday@mail.hzau.edu.cn (D.G.); luozhr@mail.hzau.edu.cn (Z.L.)

**Keywords:** persimmon, tannin, laccase, microRNA, polymerization

## Abstract

Persimmon fruits accumulate a large amount of proanthocyanidins (PAs), which makes an astringent sensation. Proanthocyanidins (PAs) are the polymers of flavan-3-ols stored in plant vacuoles under laccase activation. A laccase gene, *DkLAC2*, is putatively involved in PAs biosynthesis and regulated by microRNA (DkmiR397) in persimmon. However, the polymerization of PAs in association with miRNA397 still needs to be explored in persimmon. Here, we identified pre-DkmiR397 and its target gene *DkLAC2* in ‘Eshi 1’ persimmon. Histochemical staining with GUS and dual luciferase assay both confirmed DkmiR397-*DkLAC2* binding after co-transformation in tobacco leaves. Diverse expression patterns of *DkLAC2* and DkmiR397 were exhibited during persimmon fruit development stages. Moreover, a contrasting expression pattern was also observed after the combined *DkLAC2*-miR397 transformation in persimmon leaves, suggesting that DkmiR397 might be a negative regulator of *DkLAC2*. Similarly, the transient transformation of DkmiR397 in persimmon fruit discs in vitro also reduced PA accumulation by repressing *DkLAC2*, whereas the up-regulation of *DkLAC2* increased the accumulation of PAs by short tandem target mimic STTM-miR397. A similar expression pattern was observed when overexpressing of *DkLAC2* in *Arabidopsis* wild type (WT) and overexpression of *DkLAC2*, DkmiR397 in persimmon leaf callus. Our results revealed that the role of DkmiR397 repressed the expression of *DkLAC2* concerning PA biosynthesis, providing a potential target for the manipulation of PAs metabolism in persimmon.

## 1. Introduction

Persimmon (*Diospyros* kaki Thunb.) is a deciduous fruit tree that is believed to originate in China and is widely cultivated throughout Eastern Asia [1]. With 937,327 ha and a production of 3,342,059 t in 2020, China is the world’s largest persimmon grower, accounting for 93.2% and 78.8% of global persimmon cultivating area and output, respectively (FAOSTAT, 2022). Persimmon is the edible fruit that belongs to the *Diospyros* genus of the Ebenaceae family. Persimmon accumulates proanthocyanidins (PAs) in leaves and fruits. PAs belong to the category of secondary metabolites that play an essential role in plant survival against adverse environmental conditions [2,3,4]. These compounds provide astringency to fruits and beverages and positively impact human health [5].

Generally, a large amount of PAs (also called “condensed tannin”) accumulate in the vacuole of persimmon fruit flesh cell during its development and give an astringent taste. According to the genetic property of the natural de-astringency process, persimmon varieties are divided into two categories. Based on the relationship between astringency in persimmon fruit at harvest, the presence of seed, the flesh color, and the loss of natural astringency on the tree, persimmon genotypes were pomologically classified into two major groups as pollination-constant nonastringent (PCNA) and non-PCNA. The pollination-constant nonastringent (PCNA) type has two subcategories: Chinese PCNA (C-PCNA) and Japanese PCNA (J-PCNA), while the non-PCNA type comprise three subcategories: pollination-variant nonastringent (PVNA), pollination-variant astringent (PVA), and pollination-constant astringent (PCA). The pollination constant non-astringent (PCNA) persimmon is a mutant trait that naturally reduces its astringency throughout fruit maturation on the tree, resulting in edible fruits when fully ripening. For non-PCNA varieties, they need artificial processing or seeds to eliminate astringency before eating [6,7].

The reduction in astringency in Japanese PCNA cultivars (J-PCNA) is associated with the dilution of PAs as the fruit grows in size. In contrast, tannin cell production terminates during the early stages of fruit development, while tannin cell growth continues in non-PCNA and C-PCNA fruits until later phases. Even though both the C-PCNA and J-PCNA varieties produce fruit that loses astringency when they are ripening, the C-PCNA variety differs markedly from the J-PCNA variety in several ways [7,8]. Chinese PCNA (C-PCNA) has a considerably larger molecular weight than J-PCNA [9]. These findings suggested that the decrease in astringency in C-PCNA variety fruit could be due to soluble PAs in the flesh coagulated into insoluble status together with dilution effects of tannin.

PA precursors are transported from cytoplasm into and stored in the vacuole under membrane transporters association. Genetic studies in *Arabidopsis* have shown that PA precursors transport requires particular carrier proteins such as multidrug and toxic compound efflux protein (MATE, encoded by *TT12*), glutathione *S*-transferase (GST, encoded by *TT19*), and proton pump ATPase 10 (autoinhibited H1-ATPase isoform AHA10) that facilitate the transportation [10,11]. Laccases are another class of enzymes that play a significant role in polymerizing PA monomers in the vacuole. DkLAC1 is a critical member of the laccase family influencing PAs biosynthesis and isolated from the persimmon fruit ‘Eshi 1’ (C-PCNA) [12].

PAs are phenolic polymers resulting from polymerization of flavan-3-ol units [6], and persimmon PAs molecular weight was estimated to be around 1.38 × 10^4^ dalton. Vesicles transfer PA precursors from the endoplasmic reticulum to the vacuole [13,14], considering that the vesicles may be a new organelle “tannosome” derived from the chloroplast. Recently, a laccasel-like polyphenol oxidase FaTT10 in strawberry (*Fragaria* × *ananassa* cv. Benihoppe) is involved in the oxidative polymerization of proanthocyanidins [15]. Another finding in *Brassica napus* suggested that *BnTT10* gene is involved in producing proanthocyanidins, lignin, and seed coat pigmentation [16]. MtTT8, from *Medicago truncatula* linked and triggered regulators of anthocyanidin synthase (ANS) and anthocyanidin reductase (ANR), respectively, to modulate PA and anthocyanin production [17].

In general, microRNAs (miRNAs) are non-coding RNAs with 21 nucleotides that operate as negative regulators at the post-transcriptional level in animals and plants [18]. The bulk of plant miRNAs target structural genes or transcription factors (TFs) that regulate a wide range of biological processes, including development, primary and secondary metabolism, and defense mechanisms. The mode of regulation can be negative or positive; for example, they can encourage the synthesis of essential metabolites, inhibition of hazardous metabolites, and development of new metabolites [19,20,21,22,23,24,25,26]. In *Arabidopsis*, miR408, miR397, miR857, and miR398 target one or several members of the *Arabidopsis* LAC family, respectively, which is negatively correlated with the expression level of target gene mRNA [27]. At present, the miR397 family has also been reported in several plants, which negatively regulate the vascular lignin biosynthesis in poplar [28], and miR397 also plays a negative regulatory role in the fiber development in cotton [29].

Proanthocyanidin is recognized as a significant secondary metabolite in persimmon during fruit development. However, the role of miRNA in PA biosynthesis is still needed to be unveiled. MicroR397 was retrieved for DkmiR397 in the persimmon miRNA database, and DkmiR397 was predicted to target *DkLAC2*. The molecular dynamics of DkmiR397 and *DkLAC2* expression in Chinese PCNA (C-PCNA) persimmons fruits were studied in conjunction with changes in PAs concentration. Our findings reveal that DkmiR397 and its target gene *DkLAC2* have distinct expression patterns in persimmon fruit during development. Furthermore, our research indicated that laccase is involved in regulating proanthocyanin production in persimmon.

## 2. Results

### 2.1. Proanthocyanidins Variation during Persimmon Fruits Development

**Figure 1:**
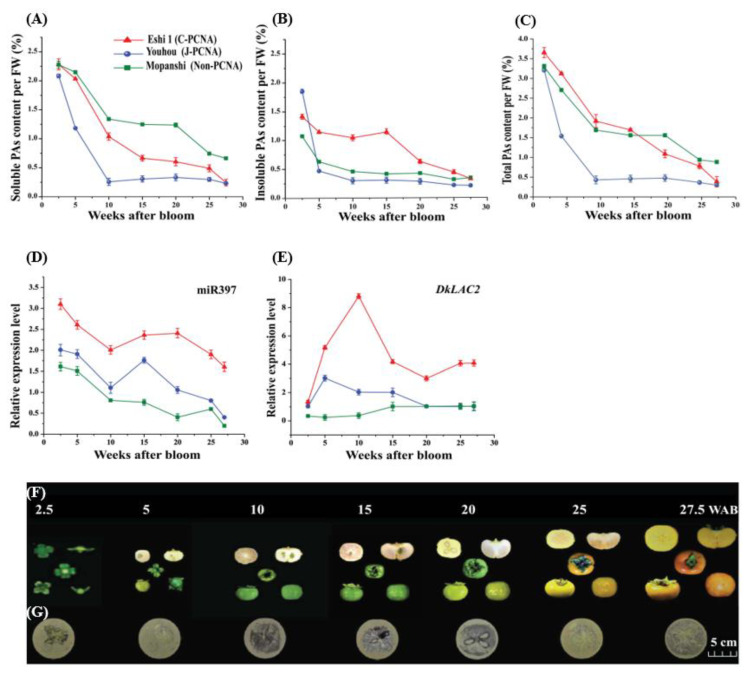
Proanthocyanidins and DkmiR397 with *DkLAC2* variation in three persimmon genotypes fruits. (**A**) Changes in soluble PAs content. (**B**) Changes in insoluble PAs content. (**C**) Changes in total PAs content. (**D**) Expression variation in DkmiR397. (**E**) Expression variation in *DkLAC2*. The ‘Eshi 1’ (C-PCNA) persimmon fruits from 2.5 WAB to 27.5 WAB were used for determining the PAs content based on the tannin printing technique (**F**,**G**). Error bars indicate SE from three biological replicates (*n* = 3).

Three persimmon varieties, ‘Eshi 1’ (C-PCNA), ‘Youhou’ (J-PCNA), and ‘Mopanshi’ (non-PCNA), were initially collected and used for measuring PAs content of their fruits. At the initial growth stage (2.5 WAB), the soluble tannin concentration of all types of persimmon fruit was found to be the highest, and it then gradually reduced with fruit development; in particular, a sharp decrease occurred in ‘Youhou’ (J-PCNA) fruits from 2.5 WAB to 10 WAB. Since 2.5 WAB, soluble tannin concentration in three persimmon genotypes fruit decreased gradually together with fruit enlargement. At 10 WAB, soluble PAs in ‘Youhou’ (J-PCNA) fruit reduced to 0.235 mg/g, and PAs terminated to accumulate since then; soluble PAs was diluted with fruits progressive development (Figure 1A). Similarly, total PAs concentration was lower in J-PCNA than that in C-PCNA and non-PCNA fruits since 5 WAB (Figure 1C).

However, in contrast, the soluble tannin concentration was retained at a high level before 25 WAB in ‘Eshi 1’ (C-PCNA) fruits, and later soluble tannin (0.645 mg/g) accounted for less than 0.2% of the fruit weight of ‘Eshi 1’, which indicated that fruits already lost their astringency. At the last experimental stage, 25 WAB to 27.5 WAB, the fruits of ‘Eshi 1’ were only slightly stained (Figure 1F,G). The soluble tannin content of ‘Mopanshi’ (non-PCNA) remained even higher and could not lose astringency even at ripening time (Figure 1A). Soluble PAs content of ‘Eshi 1’ fruits at 2.5, 5, 10, 15, 20, and 25 WAB were also visualized by the printing method. A higher concentration of insoluble PAs content in ‘Eshi 1’ (C-PCNA) fruits was observed compared to the other two varieties. Although the slight increase (0.5 mg/g) in insoluble tannin in ‘Mopanshi’ (non-PCNA) was observed after the 20 WAB (Figure 1B). These findings have suggested that 10 WAB to 20 WAB are crucial stages to ‘Eshi 1’ (C-PCNA) for soluble PAs conversion into insoluble PAs. In this study, the ‘Eshi 1’ (C-PCNA) sample was used to explore the PAs biosynthesis process.

### 2.2. Expression Patterns of MicroRNA397 and DkLAC2 in Three Persimmon Varieties

Expression patterns of the DkmiR397 and *DkLAC2* genes in ‘Eshi 1’ (C-PCNA), ‘Youhou’ (J-PCNA), and ‘Mopanshi’ (non-PCNA) persimmon fruits were analyzed using qRT-PCR (Figure 1D,E). The expression patterns of DkmiR397 and *DkLAC2* were divergent at different stages of fruit development (2.5-27.5 WAB). In ‘Eshi 1’ C-PCNA, the relative expression of DkmiR397 decreased from 2.5 WAB to 10 WAB (Figure 1D), whereas the relative expression of *DkLAC2* increased from 2.5 WAB to 10 WAB. (Figure 1E). Meanwhile, there were an obvious increase in the insoluble tannin content, of ‘Eshi 1’ (C-PCNA) fruits from 10 WAB to 15 WAB (Figure 1B). The results showed that DkmiR397 was negatively correlated with *DkLAC2* expression during the development of persimmon fruit. Lower expression levels of the *DkLAC2* gene and miRNA397 in the two types of persimmon J-PCNA and non-PCNA cultivars presented down-regulation gradually with fruit development compared to the results from C-PCNA (Figure 1D,E). Expression analysis of the structural gene that was involved in PA biosynthesis during the persimmon development stages was also assayed (Figure S1). The expression of *DkLAC2* in different tissues of three persimmon varieties was measured by qRT-PCR (Figure S2) and indicated a high transcript level of *DkLAC2* in ‘Eshi 1’ than the other varieties of J-PCNA and non-PCNA.

### 2.3. Phylogenetic Analysis and Protein Structure Prediction of DkLAC2

**Figure 2:**
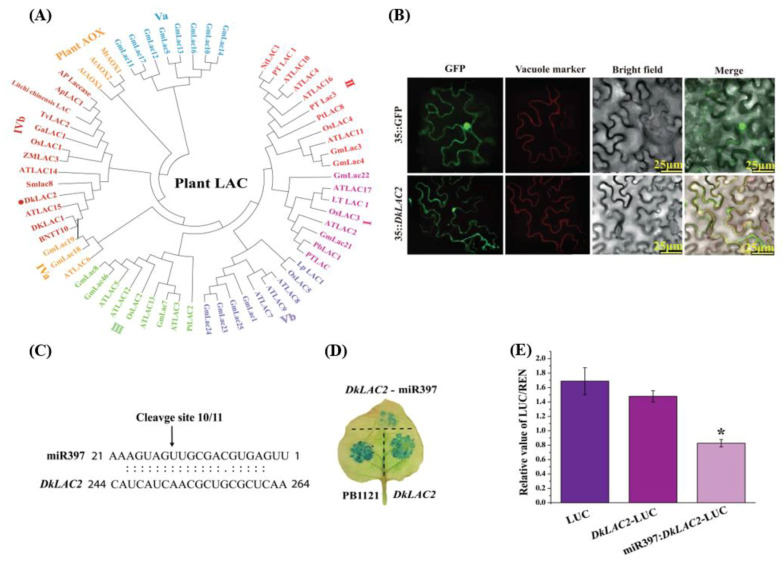
Identification and characterization of DkmiR397 and *DkLAC2*. (**A**) Phylogenetic tree of DkLAC2 homologs. The protein names from various plant species are labeled on the branches. Bootstrap replicates 3000 were used to calculate the bootstrap values. (**B**) Subcellular localization of the DkLAC2 proteins. (**C**) Target validation for DkmiR397 by RLM-5′ RACE. The cleavage sites are indicated by arrows, and the values represent the frequency of accurately sequenced clones. (**D**) GUS (histochemical β-glucuronidase) was a co-expression of DkmiR397 and its target genes in tobacco leaves via *Agrobacterium tumefaciens*-mediated transformation. (**E**) The interaction between DkmiR397 and its target genes was demonstrated using a Dual-LUC assay. Renilla (REN) luciferase activity was used to standardize relative LUC activity. Error bars indicate SEs from four biological replicates (*n* = 4). Asterisks above the bars represent values determined to be significantly different from the control by the Student’s *t*-test (* *p* < 0.05).

A phylogenetic relationship between *DkLAC2* of persimmon and other laccase proteins was performed to unveil their sequence similarity and closeness. DkLAC2 clustered together with IVb plant laccase family and presented to be closed with AtLAC15 in *Arabidopsis thaliana* (Figure 2A). The *DkLAC2* gene encoded a putative laccase protein containing a unique copper ion domain, which is the core-characteristic of the enzymes’ multi-copper oxidation family, with 356 amino acids predicted (Figure S3). The sequence similarity between DkLAC2 and AtLAC15 was 78%. The protein sequences used in this research are shown in Table S3. The DkLAC2 protein structure was designed using the Homology modeling method, and the best model was selected based on the z-score (Figure S4). The structure assessment suggested that the final three-dimensional model of DkLAC2 protein was of the best quality. The analysis of quality control through Molprobity software (http://molprobity.manchester.ac.uk/, accessed on 10 February 2021) showed that 95% of residues were in favored rotamers, and 92% were in favored regions where only 1% were poor. The highest Ramachandran plot value (89%) suggested that the structure was refined, compact, and shown in ribbon representation (Figure S5).

### 2.4. Subcellular Localization of DkLAC2 Protein

We investigated the subcellular localization of DkLAC2 in tobacco leaves using the GFP fusion construct, 35S:DkLAC2-GFP. 35S:DkLAC2-GFP recombinant plasmid co-transformed into tobacco by the *Agrobacterium*-mediated tobacco transient expression system. After 3 days, tobacco epidermal cells were observed under the confocal fluorescence microscope scanning. DkLAC2-GFP was predominantly located in the vacuole, whereas GFP (control) was uniformly distributed throughout the cell (Figure 2B). This indicated that DkLAC2 has a role that functions in the vacuole to be mediated in PAs accumulation in persimmon fruit cells.

### 2.5. Validation of the Target Genes of MicroRNA397

The secondary structure of the DkmiR397 precursor was predicted by the Mfold web-server. The sequence of the miRNA/miRNA* duplex is represented by the red box (Figure S6). RLM-5′ RACE sequencing analysis showed that the target restriction site of DkmiR397 on *DkLAC2* mRNA was at the 10-11th nucleotide at the 5′ end of the complementary sequence (Figure 2C). To verify the interaction between DkmiR397 and *DkLAC2*, we constructed transiently expressed vectors by the *Agrobacterium*-mediated method for the tobacco-transient expression system. When 35S::DkLAC2-GUS and 35S::DkmiR397-GUS vectors were injected into the tobacco leaves, the GUS signal was markedly reduced, suggesting that DkmiR397 could cut the transcript of *DkLAC2* in tobacco leaves (Figure 2D). To validate the RLM-5′ RACE results, Firefly luciferase/Renilla (LUC/REN) in the leaves inoculated with GV3101-pGreenII 0800-LUC (control) showed the same result as in the leaves inoculated with GV3101-pGreenII 0800-Target-LUC, in which the target sequence was fused upstream of the LUC gene. LUC/REN activity decreased in leaves co-transformed with GV3101-pGreenII0800-Target-LUC and GV3101-pGreen62-SKpre-miR397, which demonstrated that DkmiR397 negatively affected the expression of *DkLAC2* (Figure 2E).

### 2.6. Transient Expression MicroRNA397 and DkLAC2 in ‘Eshi 1’ (C-PCNA) Persimmon Leaves In Vivo

**Figure 3:**
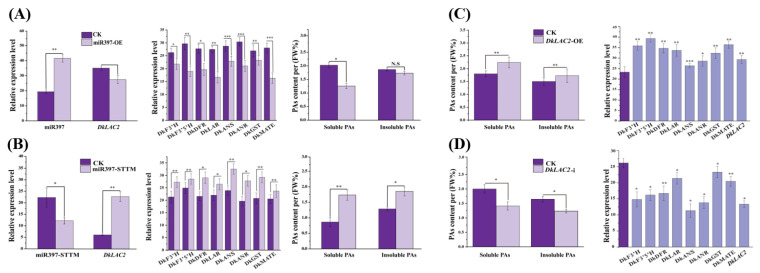
Transient expression of DkmiR397 and *DkLAC2* in ‘Eshi 1’ (C-PCNA) persimmon leaves in vivo. (**A**) Evaluation of DkmiR397, *DkLAC2*, and PA biosynthesis pathway gene transcript levels, as well as the related fluctuation in PA content, after transient overexpression of pre-miR397 at ten days after agroinfiltration. (**B**) Evaluation of DkmiR397, *DkLAC2*, and PA biosynthesis pathway gene transcript levels, and the related fluctuation in PA content, after transient of STTM397 at ten days after agroinfiltration. (**C**) Evaluation of *DkLAC2*-OE, and PA biosynthesis pathway gene transcript levels, and the related fluctuation in PA content, after transient overexpression of *DkLAC2* at ten days after agroinfiltration (**D**) Evaluation of *DkLAC2*-i, and PA biosynthesis pathway gene transcript levels, and the related fluctuation in PA content, after transient silencing of *DkLAC2*-i at ten days after agroinfiltration. F3′H: flavonoid 3′-hydroxylase; F3′5′H: flavonoid 3′5′-hydroxylase; DFR: dihydroflavonol 4-reductase; ANS: anthocyanidin synthase; ANR: anthocyanidin reductase; LAR: leucoanthocyanidin reductase; GST: glutathione *S*-transferase; MATE: multi-drug and toxic compound extrusion transporter. Errors bars indicate SEs from three biological replicates (*n* = 3). Asterisks above the bars indicate values determined by Student’s *t*-test to be significantly different from the control (* *p* < 0.05, ** *p* < 0.01, *** *p* < 0.001).

To verify the role of DkmiR397 in regulating the proanthocyanidin biosynthesis in persimmon, transient expression of DkmiR397 and *DkLAC2* were carried out in ‘Eshi 1’ (C-PCNA) persimmon leaves in vivo. Compared to CK, the expression of DkmiR397 increased after infiltration with pre-miR397 vectors, while the expression of *DkLAC2* decreased in the DkmiR397 transformed leaves (Figure 3A). After overexpressing DkmiR397, soluble tannin and insoluble tannin content decreased remarkably in persimmon leaves infiltrated with pre-miR397 vectors (Figure 3A). The transcripts level of the PAs biosynthesis pathway structural genes was reduced significantly compared with that in CK (Figure 3A). After interference with STTM-miR397 infiltrated in persimmon leaves, the expression of DkmiR397 was down-regulated, whereas the expression of *DkLAC2* increased significantly (Figure 3B). In contrast, when DkmiR397 interfered in persimmon leaves, the transcripts level of structural genes participating in PA biosynthesis elevated remarkably (Figure 3B). Thus, it indicated that DkmiR397 might negatively regulate expression of the key structural genes involved in persimmon proanthocyanidin synthesis through its cleavage on *DkLAC2*.

Further, overexpression of *DkLAC2* (OE-*DkLAC2*) and interference vector (*DkLAC2*-i) were constructed and transformed in the persimmon leaves. Compared to the control, the expression of the *DkLAC2* in transgenic lines (OE-*DkLAC2*) was markedly increased. Similar up-regulation of the structural gene in PAs biosynthesis was observed; thus, PAs content also increased (Figure 3C). In contrast, the transcript level of the *DkLAC2* decreased together with structural genes in PA biosynthesis pathway after RNAi was performed in persimmon leaves (Figure 3D). This accounted for the reason for the reduction in soluble PAs and insoluble PAs content (Figure 3D). The two sets of control experiments described above showed that *DkLAC2* enhanced the PAs pathway gene expression and resulted in promoting proanthocyanidin accumulation in ‘Eshi 1’ (C-PCNA) persimmon.

### 2.7. Transient Expression of MicroRNA397 and DkLAC2 in ‘Eshi 1’ (C-PCNA) Persimmon Fruit Discs In Vitro

**Figure 4:**
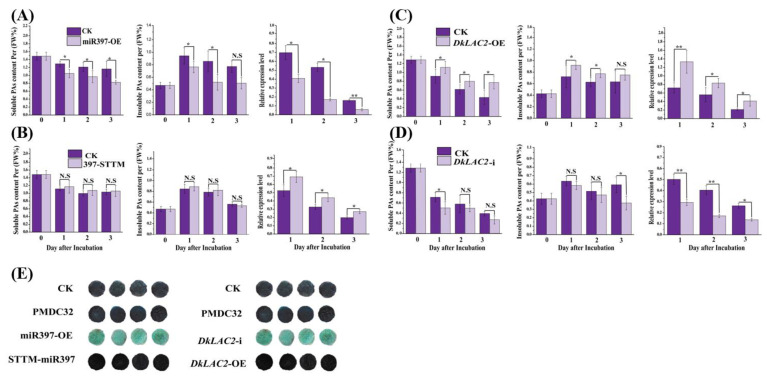
Transient expression of DkmiR397, *DkLAC2 in vitro,* and DMACA staining technique in ‘Eshi 1’ (C-PCNA) persimmon. Relative expression and PAs concentration by Folin-Ciocalteu after transformation with (**A**) DkmiR397, (**B**) STTM-DkmiR397, (**C**) *DkLAC2*-OE, (**D**) *DkLAC2*-I, and (**E**) DMACA staining of fruit discs of ‘Eshi 1’ was performed 3 days after infection. CK, blank control; pMDC32, empty vector. Error bars indicate SEs from three biological replicates (*n* = 3). Asterisks above the bars indicate values determined by Student’s *t*-test to be significantly different from the control (* *p* < 0.05, ** *p* < 0.01); N.S: non-significant.

After pre-miRNA397 was cloned and expressed in ‘Eshi 1’ (C-PCNA) fruit discs, the DMACA staining of the transformed fruit discs exhibited lighter color (Figure 4E), and it indicated that the PA content decreased correspondingly (Figure 4A). When STTM397 was conducted in fruit discs, it caused a PAs level increase (Figure 4B) and resulted in the dark color of the DMACA staining (Figure 4E). The transient overexpression of *DkLAC2* was performed in ‘Eshi 1’ (C-PCNA) fruit discs and could elevate PAs content level and cause a dark color after DMACA staining (Figure 4C,E). The phenotype is opposite to that observed for *DkLAC2*-i transformed fruit discs (Figure 4D,E). Taken together, those revealed a negative correlation between DkmiR397 and *DkLAC2* expression level, which is involved in regulating persimmon PAs accumulation.

### 2.8. Genetic Transformation of MicroRNA397 and DkLAC2 in Persimmon Leaf Callus

**Figure 5:**
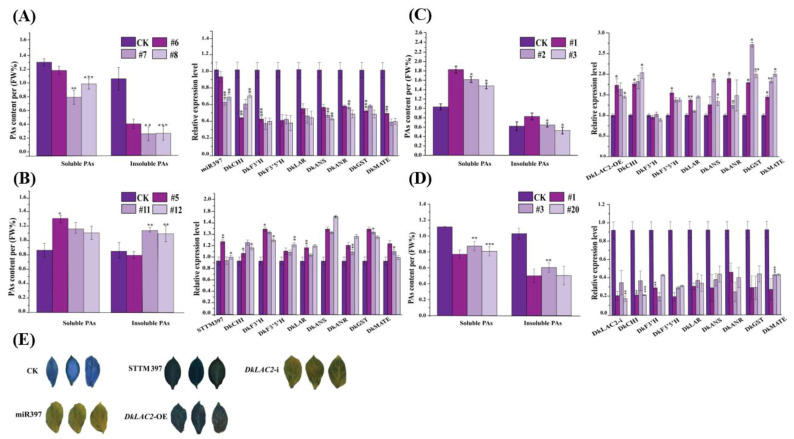
Overexpression of Pre-miR397, STTM-miR397, *DkLAC2*-OE, and *DkLAC2*-i with a stable transformation system in ‘Gongcheng Shuishi’ persimmon. Quantitative measurement of transcript level of genes in the PAs biosynthesis pathway and PAs contents variation in ‘Gongcheng Shuishi’ persimmon transgenic lines of expressing by (**A**) DkmiR397, (**B**) STTM397, (**C**) *DkLAC2,* and (**D**) *DkLAC2*-i. (**E**) The DMACA staining of regenerated transgenic lines leaves of DkmiR397-OE, DkSTTM-397, *DkLAC2*-OE, and *DkLAC2*-i. CHI: chalcone isomerase; F3′H: flavonoid 3′-hydroxylase; F3′5′H: flavonoid 3′5′-hydroxylase; ANS: anthocyanidin synthase; ANR: anthocyanidin reductase; LAR: leucoanthocyanidin reductase; GST: glutathione *S*-transferase; MATE: multi-drug and toxic compound extrusion transporter. Errors bars indicate SEs from three biological replicates (*n* =3). Asterisks above the bars indicate values determined by Student’s *t*-test to be significantly different from the control (* *p* < 0.05, ** *p* < 0.01, *** *p* < 0.001).

To identify the molecular function of DkmiR397 on PAs accumulation in persimmon ‘Gongcheng Shuishi’ (PCA) callus, we analyzed the expression pattern of DkmiR397 and the PAs content variation in three transgenic overexpression lines #6, #7, and #8 (Figure 5A). Low expression of DkmiR397 was preceded by a corresponding reduction in PAs contents and the downregulation of specific structural PA biosynthesis pathway genes according to qRT-PCR detection in three DkmiR397 transgenic lines (Figure 5A). Further, higher PAs contents in level STTM #5, #11, and #12 transgenic line was observed than that in the control (Figure 5B). The DkmiR397-STTM increased expression was preceded by an approximately equal increase in the expression levels of *CHI* (chalcone isomerase gene), *LAR* (leucoanthocyanidin reductase gene), and *F3*′*5*′*H* (flavanone 35′-hydroxylase gene) according to qRT-PCR analysis of these three DkmiR397-STTM transgenic lines (Figure 5B). Altogether, these results suggested that DkmiR397 negatively regulates PA accumulation in persimmon by directly modulating *DkLAC2*.

Further, we analyzed that the molecular function of *DkLAC2* on PAs accumulation in persimmon ’Gongcheng Shuishi’ (PCA) callus was transformed with *DkLAC2* under the transcriptional control of the CaMV35S promoter. A total of 18 transgenic calluses of the *DkLAC2*-OE were obtained. Finally, three positive regenerated transgenic seedlings *DkLAC2*-OE #1, #2, and #3 were identified via PCR confirmation converted by CaMV35S-sense *DkLAC2*, which were chosen for further experiments based on the minor increase in PA content (Figure 5C). DMACA staining was used to detect PAs contents among transgenic and wild-type plants (Figure 5E). Additionally, we analyzed the expression pattern of silence (*DkLAC2*-i) in three lines #1, #3, and #20, which were chosen for the expression level of PA content (Figure 5D). The qRT-PCR results suggested that the low expression of *DkLAC2*-i was preceded by a reduction in the expression levels of PA biosynthesis pathway-specific structural genes and led to PAs contents reduction (Figure 5D).

### 2.9. Genetic Transformation of DkLAC2 in Wild-Type Arabidopsis

**Figure 6:**
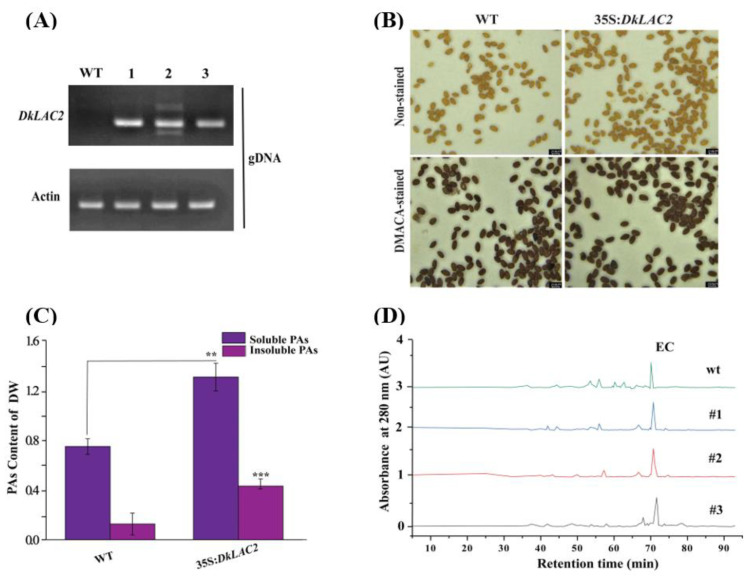
Functional analysis of *DkLAC2* via transformation in *Arabidopsis*. (**A**) Semi-quantification of *DkLAC2* in WT *Arabidopsis* and transgenic lines. (**B**) Seed coat pigmentation before and after staining colorations by DMACA reactions comparison between wild-type *Arabidopsis* seeds and T1 Arabidopsis transgenic lines derived from *DkLAC2* transformation. Bars = 250 µm. (**C**) PAs content of WT and 2 × 35S:*DkLAC2 Arabidopsis* transgenic plants seeds. (**D**) HPLC analysis of PAs monomers in *Arabidopsis* transgenic line compared to WT. Data represent the mean of three biological replicates, and error bars represent the standard deviation of three replicates (*n* = 3). Asterisks above the bars indicate values determined by Student’s *t*-test to be significantly different from the control (** *p* < 0.01, *** *p* < 0.001).

We transformed 2 × 35S:pre-DkLAC2 into WT *Arabidopsis* ecotypes: Columbia (Col-0). The complementary phenotype of *Arabidopsis* seeds was further verified via DMACA staining. Seed color did not show significant variations between transgenic and wild-type lines. After this, a positive detection test was carried out. As expected, the target gene *DkLAC2* was detected to be significantly expressed in transgenic lines of *Arabidopsis* (Figure 6A). Brownish pigmentation showed that PAs accumulated in the seed coats expressing 2×35S:DkLAC2 T1 transgenic lines (Figure 6B). It was confirmed that PAs contents increased remarkably than in the wild-type by quantification measurement (Figure 6C). These results showed that the *DkLAC2* are functionally involved in PAs accumulation.

HPLC analysis of PA subunits after acid catalysis in the presence of excess phloroglucinol was carried out based on Akagi et al. (2010) [30]. A lower epicatechin (EC) peak was found in WT seed (Figure 6D, Figure S7). These findings suggested that the increase in total soluble PAs was mostly caused by an increase in epicatechin content in the *DkLAC2* transgenic lines.

## 3. Discussion

In the past decade, fungal laccase biology has been the subject of intensive research. However, analyses of laccase in the higher plant’s physiological function are relatively rare. Laccase is a multi-copper oxidase that acts as a catalyst one-electron oxidation of a variety of phenolics [31,32,33,34]. Laccase gene (*DkLAC2)* in persimmon (*Diospyros kaki*) has a long-conserved sequence that putatively binds to copper ion in a unique way. *DkLAC2* might act as a downstream structural gene that plays a role in polymerizing proanthocyanidin. Phylogenetic analysis suggested that DkLAC2 laccase protein that has a function similar to *Arabidopsis*. Commercial persimmon varieties are perennial and hexaploidy, and a lack of genome sequence information makes it difficult to elucidate the mechanisms influencing persimmon PAs metabolism. Mostly, there are some reports on the miR397-targeted laccase gene family in *Arabidopsis*, poplar, tomato, cotton, strawberry, grapes, and other plants. MiR397 generally acts as a negative regulator to inhibit the expression of the target gene *LAC*. In poplar, miR397 inhibits the expression of *LAC* at the post-transcriptional level, which hinders lignin synthesis [28]. Laccase function was discovered in the sap of the Japanese lacquer tree *Rhus vernicifera* [35], while in the plant, miR397 regualtes lignin and proanthocyanidin and the oxidative polymerization of monolignols via modulating laccase [36,37].

Proanthocyanidin monomers and polymerization are most likely to occur after following passage into the vacuole [38]. The exact process of monomer condensation to generate the proanthocyanidin polymer is unknown, even though the TT10 laccase-like polyphenol oxidase has been involved in the oxidative polymerization of epicatechin and other flavonoids in *Arabidopsis thaliana* [39]. The biosynthesis and metabolism profile of PAs in persimmon is essentially not clear. RT-PCR was used to determine the level of expression of early PA biosynthesis-related genes and late biosynthesis-related genes in the fruit flesh of three different varieties ‘Eshi 1’ (C-PCNA), ‘Youhou’ (J-PCNA), and ‘Mopanshi’ (non-PCNA). From 10 WAB, the expression of early PA biosynthesis-related genes was instantaneously down, whereas the expression of late PA biosynthesis-related genes was increased in some varieties of persimmon (Figure S1), as described in the previous report [6]. At 10 WAB, the expression of *DkANS*, *DkANR*, *DkGST,* and *DkLAC2* kept a relatively high level in the C-PCNA variety (Figure S1). In this study, the *DkLAC2* gene was obtained by the homology-based cloning method. DkLAC1 is phylogenetically related to the known enzyme *AtLAC15*, which involves oxidative polymerization of PAs in *Arabidopsis* [12]. Bioinformatics analysis suggested that *DkLAC2* has a preserved sequence unique to laccase protein and binds to copper ions. A phylogenetic study of laccase genes from other species and persimmon had shown that *DkLAC2* clusters are present in the fourth laccase family of plants, which is closest to ATLAC15/tt10 in the *Arabidopsis* laccase gene family [40]. Subcellular localization of ADE/LAC-YFP subcellular was observed around the edges of the red-stained Lysotracker vacuoles, as well as within the plasmolysed epidermal cell vacuoles [41]. Based on PSORT program bioinformatics analyses, *DkLAC1* was predicted to be localized in vacuoles, where PAs aggregate and the polymerization reaction occurs [12]. Recently, LAC14-4 was visualized in vacuoles and the endoplasmic reticulum in tobacco leaves [42]. DkLAC2 can also be detected in the vacuole of epidermal cells in a tobacco leaf (Figure 2B).

In conclusion, many studies have proved that microRNA397 regulates the target gene *LAC* at the post-transcriptional level, which is consistent with our findings through qRT-PCR analysis of expression pattern, RLM-5′RACE, GUS histochemical staining followed by double luciferase activity (LUC) experiment. Laccase1-like FaTT10 was reported to be involved in the proanthocyanin biosynthesis in strawberries [15]. Our results showed that DkmiR397 negatively correlates with the pattern of expression of the target gene *DkLAC2*, which is consistent with the interaction between the miRNA and the target gene. During the fruit developmental stages of 10 WAB to 25 WAB, the relative expression of DkmiR397 negatively correlated with *DkLAC2* expression (Figure 1F,G); this suggested that DkmiR397 negatively regulates PAs accumulation (Figure 1C,F).

We further examined the role of DkmiR397 and *DkLAC2* in the metabolism of persimmon proanthocyanidin through the proven method of transformation on persimmon leaves [43]. The results of stable transformation with DkmiR397 and *DkLAC2* in persimmon leaf callus was coincident with the output from transient transformation of DkmiR397 and putative target gene *DkLAC2* in persimmon leaves or fruit disc.

**Figure 7:**
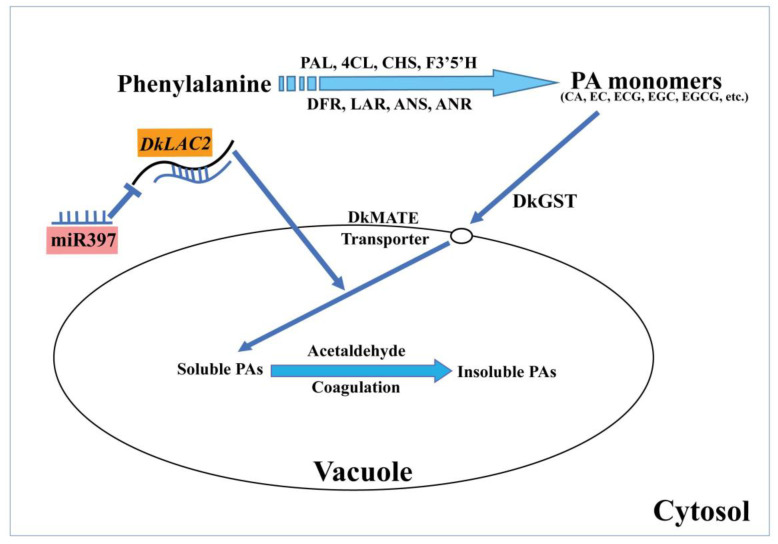
A working model of DkmiR397 and *DkLAC2* regulates PA biosynthesis in C-PCNA persimmon fruits. PAL: phenylalanine ammonia-lyase; 4CL: 4-coumarate: coenzyme A ligase; CHS: chalcone synthase; F3′5′H: flavanone 3′5′-hydroxylase; DFR: dihydroflavonol 4-reductase; LAR: leucoanthocyanidin reductase; ANS: anthocyanidin synthase; ANR: anthocyanidin reductase; GST: glutathione *S*-transferase; MATE: multi-drug and toxic compound extrusion transporter; CA: catechin; EC: epicatechin; ECG: epicatechin-3-O-gallate; EGC: epigallocatechin; EGCG: epigallocatechin 3-O-gallate.

Based on our findings and recent research, we proposed a hypothesis model that integrates structural genes, transporter, DkmiR397, and *DkLAC2* that regulates in the PA biosynthesis in C-PCNA persimmon fruit (Figure 7). The findings explicitly suggested that DkmiR397 repressed *DkLAC2* expression, which may contribute to PA regulation.

## 4. Materials and Methods

### 4.1. Plant Materials

Both persimmon fruit and leaf samples were obtained from the persimmon orchard in Huazhong Agricultural University, Wuhan, China. Persimmon fruits (*Diospyros kaki* Thunb.; 2n = 6x = 90) of ‘Eshi 1’ (C-PCNA), ‘Youhou’ (J-PCNA), and ‘Mopanshi’ (Non-PCNA) were collected at 2.5, 5, 10, 15, 20, 25, and 27.5 weeks after bloom (WAB). Three biological replicates were collected from three different individuals, with each treatment including 10-15 fruits. After sampling, all samples were promptly frozen in liquid nitrogen and stored at -80 °C in a freezer. Frozen samples were used directly to extract RNA or lyophilized for the extraction of PAs. ‘Gongcheng Shuishi’ (PCA) in vitro culture seedlings on the medium of MS (Murashige and Skoog) (1/2N) were under a 12 h light/dark cycle in a 24 °C chamber. The three positive transgenic persimmon seedlings of *DkLAC2*-OE (*DkLAC2*-1, *DkLAC2*-2, and *DkLAC2*-3), *DkLAC2*-i (*DkLAC2*-1, *DkLAC2*-3, and *DkLAC2*-20), DkmiR397-OE (DkmiR397-6, DkmiR397-7, and DkmiR397-8), and DkmiR397-short tandem target mimic (STTM) (397-STTM-5, 397-STTM-11, and 397-STTM-12) were propagated for further analysis. For GUS: β-glucuronidase (GUS), Firefly luciferase (LUC), subcellular localization analysis, tobacco (*Nicotiana *benthamiana**), and *Arabidopsis thaliana* (WT) were grown in the incubator at a room temperature of 16-h light/8-h dark.

### 4.2. Determination of Proanthocyanidin Content

The precise determination of soluble and insoluble PAs in flesh pulp and leaves was performed by the Folin-Ciocalteu method. Soluble PAs were extracted at room temperature from 50 mg of fine crushed frozen samples in 25 mL of 80% methanol. According to the previous report, insoluble PAs were extracted from the residue in 25 mL of 1 percent (*v*/*v*) HCl-methanol for 1 h at 60 °C [44]. DMACA (p-dimethylaminocinnamaldehyde) staining is used to determine and visually represent the PAs content in persimmon fruit discs [45]. According to Eaks (1967) [46], the printing method was used to evaluate the soluble PAs content of the fruit during the developing stages. Darker blue color visualization means a higher PAs contents due to the soluble tanin reaction with FeCl_2_.

### 4.3. Total RNA Extraction and Quantitative Reverse Transcription PCR

Total pulp RNA was extracted using the RNAplant Plus Reagent kit (Tiangen, Beijing, China) based on the manufacturer’s protocol. Three biological replicates of each sample were kept at -80 °C and used for the extraction of RNA. The purity and integrity of the total RNA were determined by a NanoDrop 2000 spectrophotometer (Thermo Scientific, Madison, WI, USA) and gel electrophoresis using the PrimeScriptTM RT Kit with gDNA Eraser (TaKaRa, Dalian, China) according to the manufacturer’s instructions; 2.0 µg of each RNA sample was used to generate first-strand cDNA for gene isolation. For miRNA reverse transcription, Mir-XTM miRNA first-strand synthesis and the TB Green Real-Time PCR (qPCR) kit were used (TaKaRa, China). The QuantStudio 7 Flex Real-Time PCR system (Applied Biosystems, Thermo Fisher Scientific, Singapore) was applied to perform quantitative reverse transcription PCR (qRT-PCR). The qRT-PCR patterns were performed at 95 °C for 5 min, followed by 40 cycles of 95 °C for 10 s, 58 °C for 30 s, and 72 °C for 20 s. As an internal control U6 gene, DkActin (accession no. AB473616) was used. The forward miRNA primer was designed using the fasta sequence, and universal primer was used as a reverse primer (Table S1).

### 4.4. Cloning of the DkLAC2 Gene

There is a small RNA library of ‘Eshi 1’ (C-PCNA) fruit at 15 WAB and 20 WAB, from which some candidate miRNAs were proposed to participate in the PAs metabolism pathway and natural astringency regulation network of C-PCNA by our lab previous work [47]. In addition, the transcriptome profiling of the ‘Eshi 1’ (C-PCNA) fruits of 10 WAB and 20 WAB were characterized [48]. UniGene68756_All (*DkLAC2*) were predicted and screened out as candidate target genes of DkmiR397. Laccase gene *DkLAC2* was cloned by RACE (rapid amplification of cDNA ends) using the SMARTer^®^ RACE 5′/3′ kit (TaKaRa, Dalian, China) based on the original UniGene68756_All sequence. The primer sequences used for RACE and cloning are listed in Table S2.

### 4.5. Bioinformatics Analysis of DkLAC2 Gene

The deduced amino acid sequences of homologous genes of 18 other species were obtained from the National Center for Biotechnology Information (NCBI) database. Multiple protein sequence alignments were conducted using DNAMAN version 8.0 (Lynnon Biosoft). A phylogenetic tree was built through MEGA 7.0 [49]. The ProtParam tool was performed to obtain the derived LAC amino acid sequences (http://www.expasy.org, accessed on 10 July 2019). To obtain an overview of the three-dimensional structure of the DkLAC2 protein, we used the Swiss Model Portal to perform molecular modeling. The selection of the best model was conducted based on QMEANscore. Model quality assessment analysis was carried out by SAVES (https://saves.mbi.ucla.edu/, accessed on 2 February 2021). The three-dimensional arrangement of amino acids was visualized through UCSF Chimera (https://www.cgl.ucsf.edu/chimera/, accessed on 31 January 2021).

### 4.6. Computational Prediction of DkmiR397 Target Gene and miRNA Precursor Secondary Structure

Based on the miRNA database (accession number: SRP050516) [47] and transcriptome database [48] and the psRNATarget online software [50], those databases and software were used to predict the target-targeted relationship between DkmiR397 and the potential target gene *DkLAC2*. The secondary structure of miRNA precursors was built with the RNA Folding Form tool [51].

### 4.7. RNA Ligase-Mediated 5′-RACE Verification

RNA ligase-mediated rapid amplification of 5′ cDNA ends (RLM-5′ RACE) was performed to map the cleavage sites of the target transcripts by using the GeneRacer kit (Invitrogen, USA). At 15 °C overnight, three replicates of l µg total RNA isolated from persimmon fruit samples were ligated with 5′ RACE RNA adaptors. Gene-specific primers (GSP) (Table S1) were designed to conduct 5′ RACE PCR. The PCR fragments were cloned into the pEASY-Blunt Simple vector (TransGen Biotech, Beijing, China) and subjected to sequencing.

### 4.8. Histochemical GUS Assay

To confirm the results of GUS histochemical staining for DkmiR397 and *DkLAC2*, specific primers with restriction sites were designed upstream and downstream of the ORF (open reading frame) of *DkLAC2*. *Xba* I-F and *Bam*H I-R were used as the forward primer and reverse primer (the reverse primer did not contain a termination codon). PBI121 carrying the GUS reporter gene was used as a control vector. After 3 days of tobacco injection, the leaves were collected and immersed in GUS infection solution for 15 min of vacuum infiltration until the dye solution completely infiltrated into the leaf tissue. After vacuum infiltration, it stood in the dark for 12 h under a constant temperature of 37 ℃ in the new infection solution, and then we decolorized it with 75% ethanol. We replaced the ethanol solution after every 12 h until the leaves were completely decolorized. The main reagents required for GUS staining solution were: X-Gluc solution 1mmol/L, 100 mmol/L phosphoric acid buffer (pH = 7.0), 0.1% Triton X-100, 10 mmol/L Na_2_EDTA, 0.5 mmol/L potassium ferricyanide, 0.5 mmol/L potassium ferrocyanide, and 20% methanol. A Canon 550D camera with a 100 mm magnifier was used to capture photos of dyed tissue.

### 4.9. Dual-Luciferase Assay

The *Agrobacterium*-mediated GV3101 containing pGreenII0800-Luc plant expression Luciferase Report vector was infected with *Nicotiana* *benthamiana* leaves to characterize whether *DkLAC2* was cleaved by pre-miR397 or not. The open reading frame (ORF) of *DkLAC2* without the termination codon was fused to pGreenII800-Luc after endonuclease digestion with *Kpn* I/*Bam*H I Primer shown in Table S1. To detect the activity of Firefly Luciferase (LUC) and Renilla Luciferase (REN), the Dual-Luciferase Reporter Assay System kit (Promega, Madison, WI, USA) was used.

### 4.10. Subcellular Localization of DkLAC2

To confirm the subcellular localization of DkLAC2, we constructed the fusion constructs 35S:DkLAC2-GFP and the complete open reading frames (ORFs) of *DkLAC2* without the termination codon, and they were amplified using the primers (Table S1). The 35S-GFP vector was used as a positive control. The heat shock method was used to transmit the fusion and control plasmids into *Agrobacterium* tumefaciens strain GV3101, which was subsequently transformed into the leaves of 6-week-old *Nicotiana* *benthamiana*. A needle-free syringe penetration with a solution of 10 mM 2-morpholinoethanesulfonic acid (MES), 10 mM magnesium chloride (MgCl_2_), and 150 mM acetosyringone (C_10_H_12_O_4_) at pH 5.6 was performed to penetrate *Nicotiana* *benthamiana* leaves. The fluorescent signals were observed using a Nikon Eclipse 90i fluorescence microscope three days later after infiltration.

### 4.11. Transient Genetic Transformation in Persimmon Leaves

Transient over-expression and silence-expression approaches were applied to investigate the role of DkmiRNA397 and *DkLAC2* in the regulation of proanthocyanin synthesis in persimmon leaves. Full-length pre-miRNA397 and *DkLAC2* gene sequences were transferred into the pMDC32 plant expression vector through homologous recombination, as described by Mo et al. (2016) [52], and the pMDC32 vector containing GFP was used as a positive control. All the over-expression and silence-expression primers are listed in Table S1. The vectors were introduced into persimmon leaves using a previously published *Agrobacterium*-mediated technique [43].

The short tandem target mimic (STTM) of the miRNA397 (STTM397) module was inserted into the pMDC32 vector between the 2 × 35S promoter and the 35S terminator using the reverse PCR method [53]. The *Agrobacterium* supernatant was collected after centrifugation to obtain a detection limit (OD) at 600 nm of 0.75. The infected leaves were grown on the tree before relative expression analysis and PAs content determination. One-hundred mg tissues from each infiltrated leaves were taken for PAs and expression detection; at ten days after injecting these constructions into leaves, each treatment included three biological duplicates with every ten leaves.

### 4.12. Transient Transformation in Persimmon Fruit Discs

The role of DkmiRNA397 and *DkLAC2* in the regulation of proanthocyanin synthesis in persimmon fruit disc in vitro was investigated by applying a transient over-expression and silence-expression approach. An agrobacterium infection solution was used to transform the 15 WAB ‘Eshi 1’ (C-PCNA) fruit discs with a diameter of 2 cm and a thickness of 0.4 cm for 30 min. Fruit discs were then placed in tissue culture dishes on filter paper moistened with MS liquid medium and located in an incubator at 24 °C for 3 days. Three replicates were used for each treatment, including *DkLAC2* and DkmiR397 constructs and an empty vector infection. We dyed the persimmon fruit discs in DMACA solution, soaked them in 30% glacial acetic acid and ethanol solution for 12–20 h, and then rinsed them 2–3 times with 75% ethanol. High-resolution pictures were captured using a Canon 550D camera. Fruit discs were dried on a sterilized filter paper, frozen in liquid nitrogen, and then stored at −80 °C for further analysis [54].

### 4.13. Stable Genetic Transformation of DkLAC2 and DkmiR397 in Persimmon

To examine the effects of overexpression (*DkLAC2*-OE), silencing (*DkLAC2*-i), overexpression (DkmiR397-OE), and STTM-miR397 in leaf discs of ‘Gongcheng Shuishi’ (PCA) persimmon transformation was performed by *A. tumefaciens* according to the reported method [55]. In brief, the leaf discs (8 mm in diameter) prepared from ‘Gongcheng Shuishi’ shoot leaves were pre-cultivated on the MS (1/2N) medium containing 1 μM zeatin and 1 μM NAA at 28 °C in darkness. The leaf was infected with *A. tumefaciens*, and the callus tissue was co-cultured on an MS (1/2N) solid culture medium containing 50 mg/L kanamycin as a select antibacterial agent.

### 4.14. Transformation of DkLAC2 in Arabidopsis Plants

The floral dip method [56] is the reliable and most extensively utilized protocol for creating transgenic *Arabidopsis* plants. Based on this transformation approach, the *A. tumefaciens* GV3101 line containing 2 × 35S:DkLAC2 was introduced into wild-type (WT) *Arabidopsis thaliana*. The 2 × 35S:DkLAC2 line seeds were germinated on MS media with 6% sucrose and 50 mg/L kanamycin and then transplanted into a culture substrate to produce T1 seeds.

### 4.15. HPLC Analysis of PA Subunits

According to Akagi et al., (2010) [30], soluble PAs were obtained from 10 mg of area-based dry samples in 1 mL of 70% acetone containing 0.1% ascorbic acid for 24 h at room temperature. In the presence of excess phloroglucinol, the supernatant was used for acid-catalyzed cleavage of the PAs. The PA extract powder was subjected to acidic conditions to cleave the structure of PA subunits [30], followed by functionalization with excessive phloroglucinol, as reported by [57] with minor modifications.

On a Waters HPLC system, 10 μL aliquots of the above sample were added (Waters 1525 Binary HPLC pump equipped with 2998 PDA and 2424 ELS detectors). HPLC separation was performed to use a reversed-phase C18, 5 m, 4.6 × 250 mm column with solvents A (0.2 percent formic acid) and B (methanol) with the following elution profile: 1% B for 20 min, then 15.5% for 35 min, then 45% for 50 min, followed by 15 min of washing with 100% B and a return to the original circumstances (1% B). Detection was carried out at 30 °C with a flow rate of 1 mL min^−1^ at A280 in this experiment.

## 5. Conclusions

Persimmon fruits accumulate a large amount of proanthocyanidin (PAs). MicroRNA397 was predicted to target *DkLAC2*, which is putatively mediated in PAs accumulation in C-PCNA persimmon. Expression of microRNA397 exhibits a negative correlation to the transcripts level changes of *DkLAC2* in three persimmon genotypes. There are cleavage sites of DkmiR397-targeted *DkLAC2* at 10-11 nt sites, which was validated by histochemical staining of GUS and sequencing. Based on the PAs content variation and expression of PAs biosynthesis-related genes and *DkLAC2* with microRNA397 after the transient transformation in leaves and fruit discs of persimmon, together with stable transformation in persimmon leaf callus and Arabidopsis, DkmiR397 appear to serve as a negative regulator of *DkLAC2* and PA biosynthesis. These findings will enhance our understanding of the PA metabolic pathways and may provide potential for engineering plant fruits PAs production with lower PAs concentration.

## Data Availability

The data supporting the findings of this study are available within the article and Supplementary Materials.

## Supplementary Materials

The following are available online at https://www.mdpi.com/article/10.3390/ijms23063200/s1.
