# Supplementary material for: DkmiR397 Regulates Proanthocyanidin Biosynthesis via Negative Modulating DkLAC2 in Chinese PCNA Persimmon
Source: Int J Mol Sci. 2022 Mar 16;23(6):3200. doi: 10.3390/ijms23063200 (PMC8951489; doi:10.3390/ijms23063200)
Supplement: Supplementary file 1 [file ijms-23-03200-s001.zip › Supplementary Figure S1-S6.pdf]

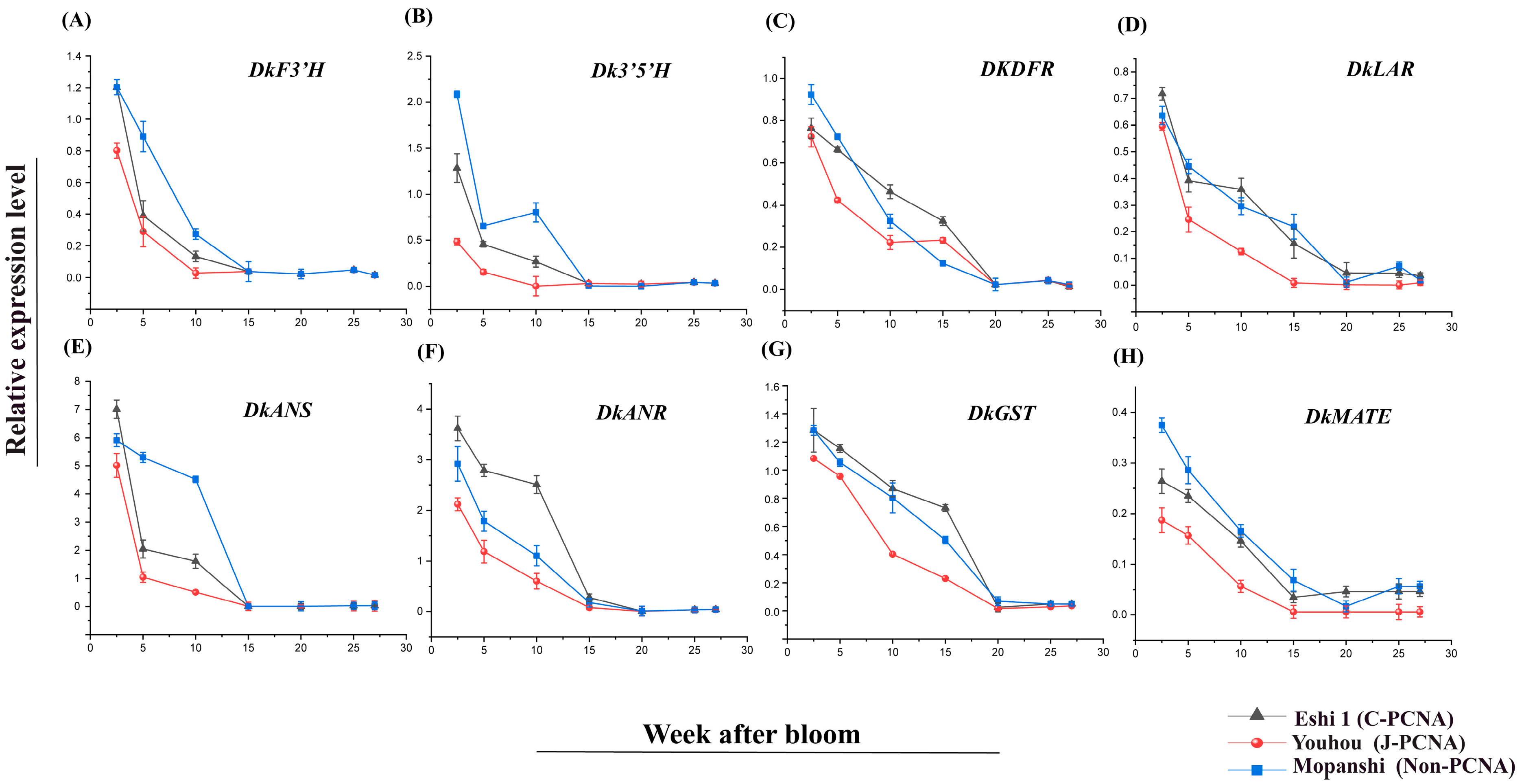

(Supplementary Figure S1a): Expression analysis of the structural gene during the persimmon development phase in three varieties.

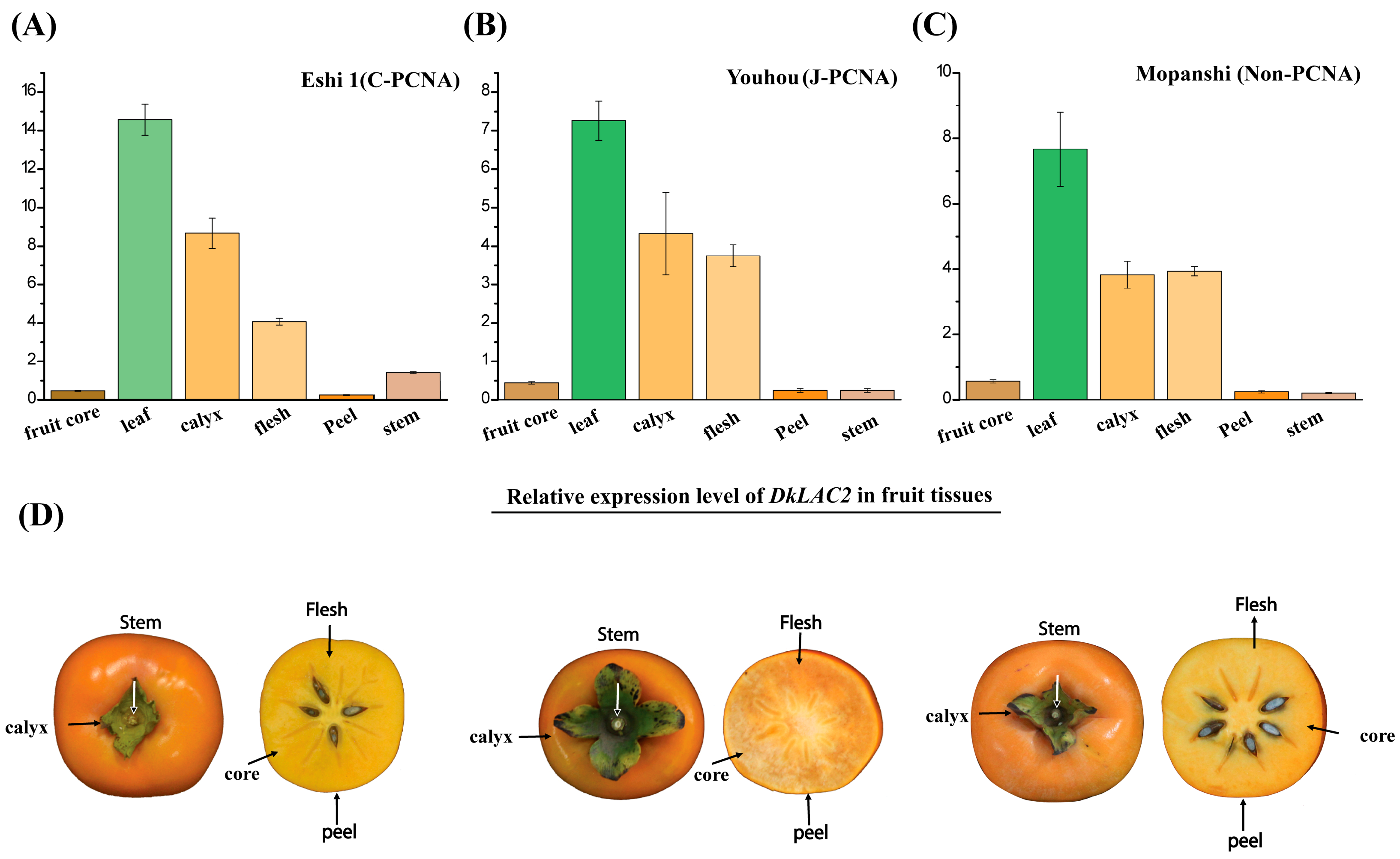

(Supplementary Figure S1b): Expression of *DkLAC2* in different tissues of three persimmon varieties.

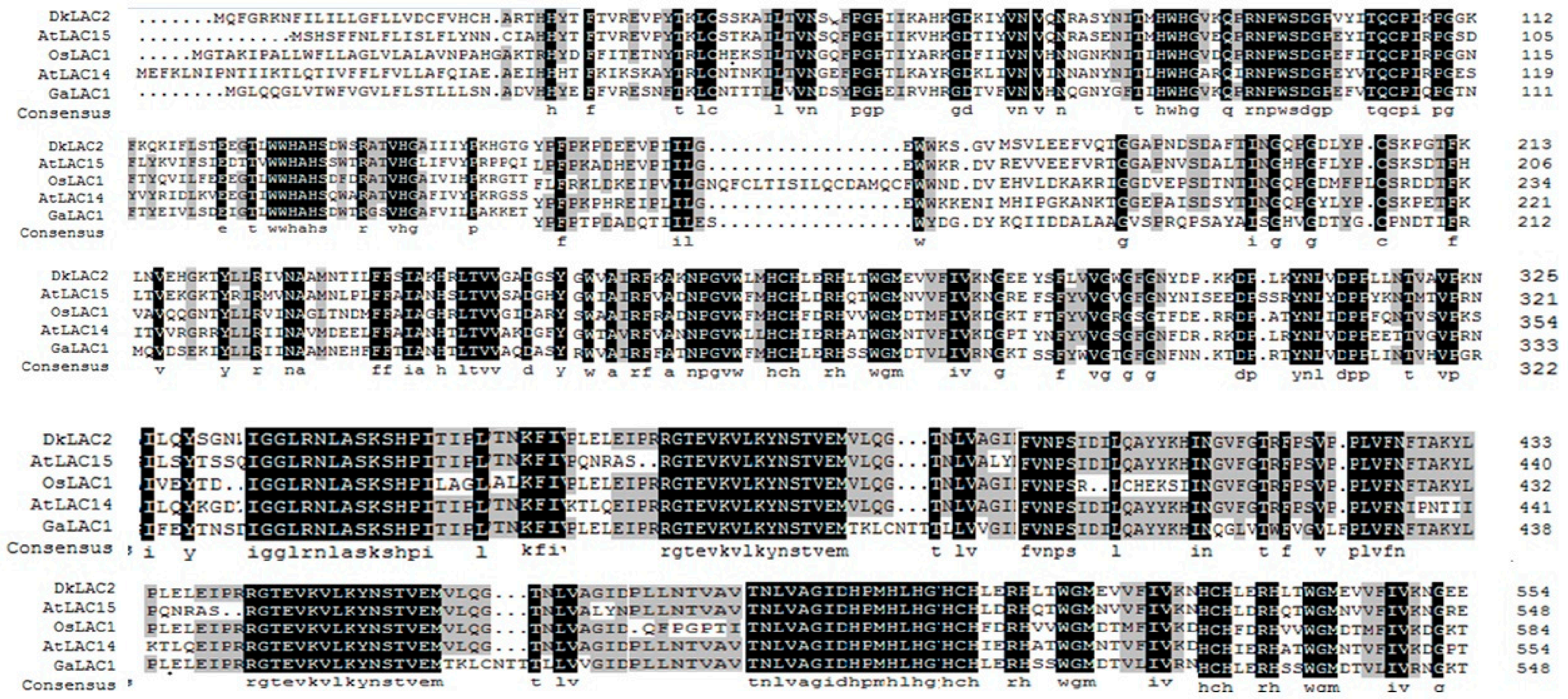

(Supplementary Figure S2): DNAMAN was used to generate a multiple sequence alignment assessment. LACs were obtained from NCBI; Persimmon DKLAC2 proteins and *ATLAC15* similarity 71.37%.

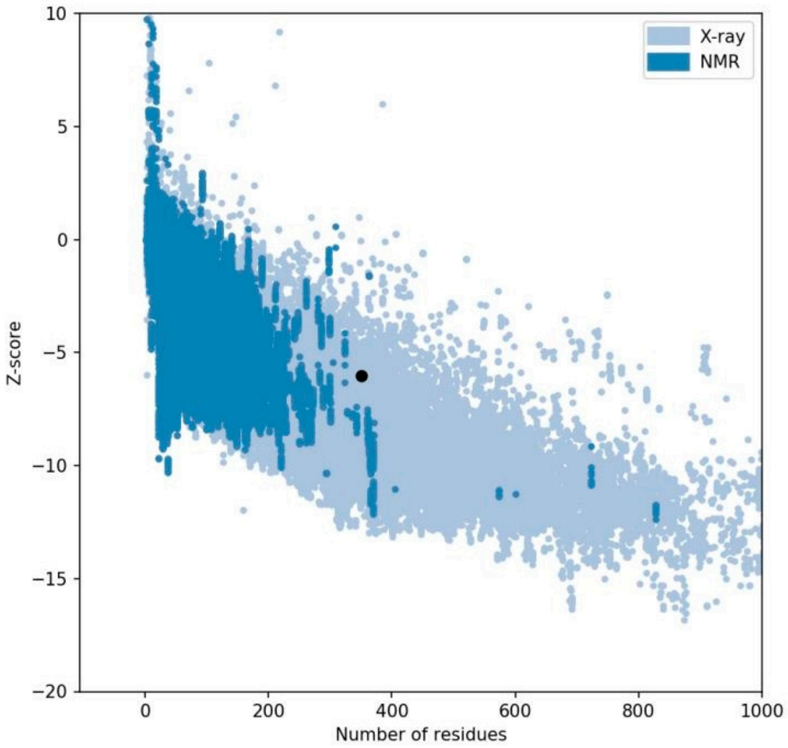

(Supplementary Figure S3): The *Z*-score represents the model quality score of predicted protein. Black dot indicating the score of protein model.

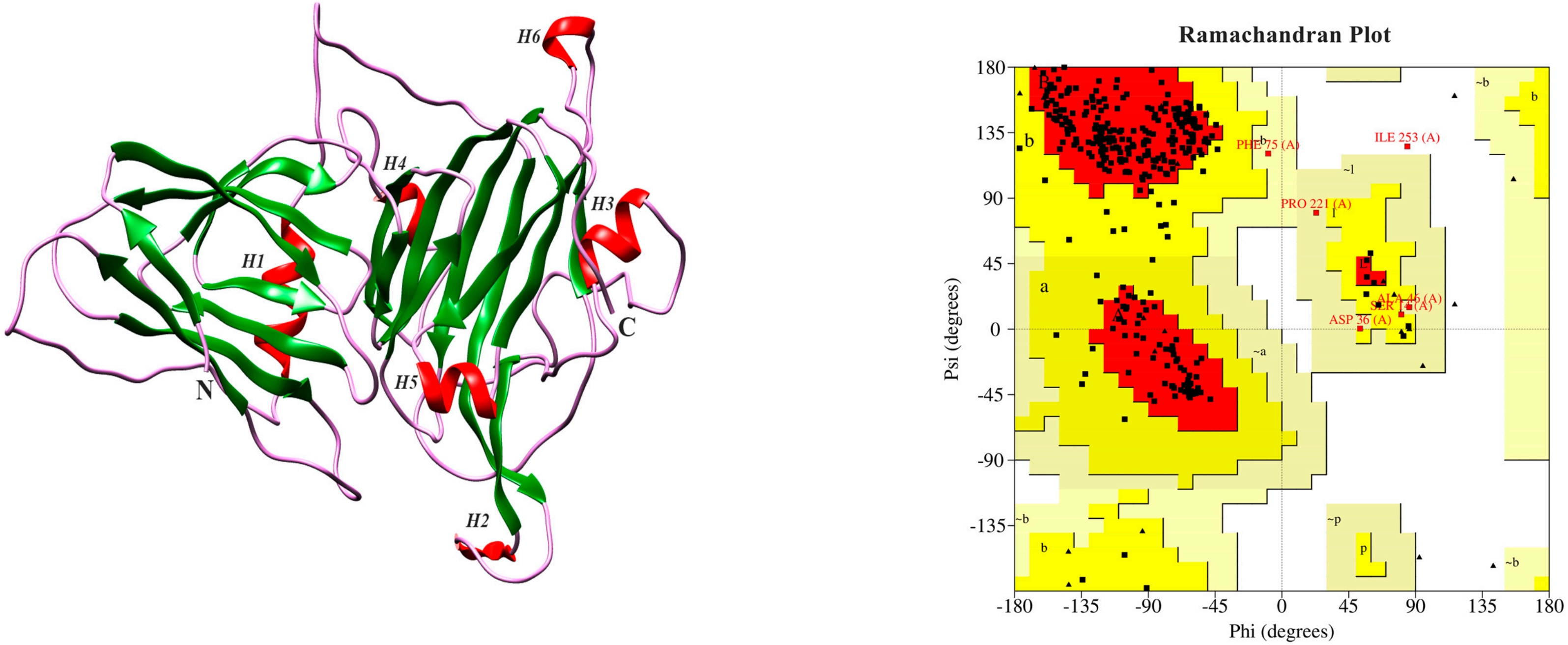

(Supplementary Figure S4): (A) Ribbon representation of DkLAC2 protein Red: Helices (H) and Green: β-Sheets and pink: connecting loops (B) Ramachandran plot suggesting the good quality of model. Red dots are indicating the distribution of residues. The first quadrant and third quadrant represent the allowed region while second and fourth quadrant indicating the disallowed region

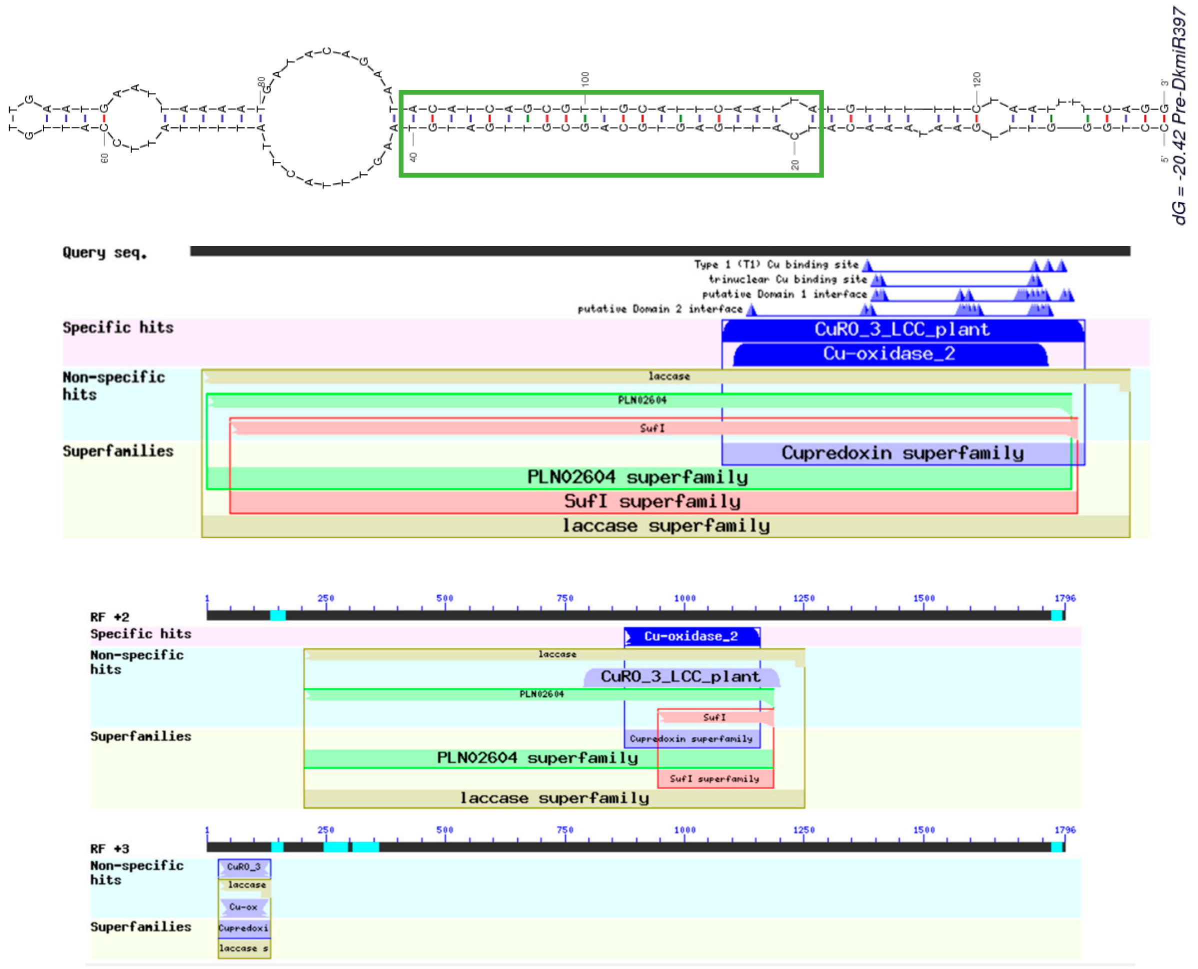

(Supplementary Figure S5): (A) The secondary structure of DkmiR397 precursor. The mature sequence in green box. (B)Gene structure of *DkLAC2*.It has typical copper ion domain and his rich conservative domain, which are typical characteristics of multi copper oxidation family. Sequence analysis showed that *DkLAC2* included 1794 BP.(C) Predicted 356 amino acids.

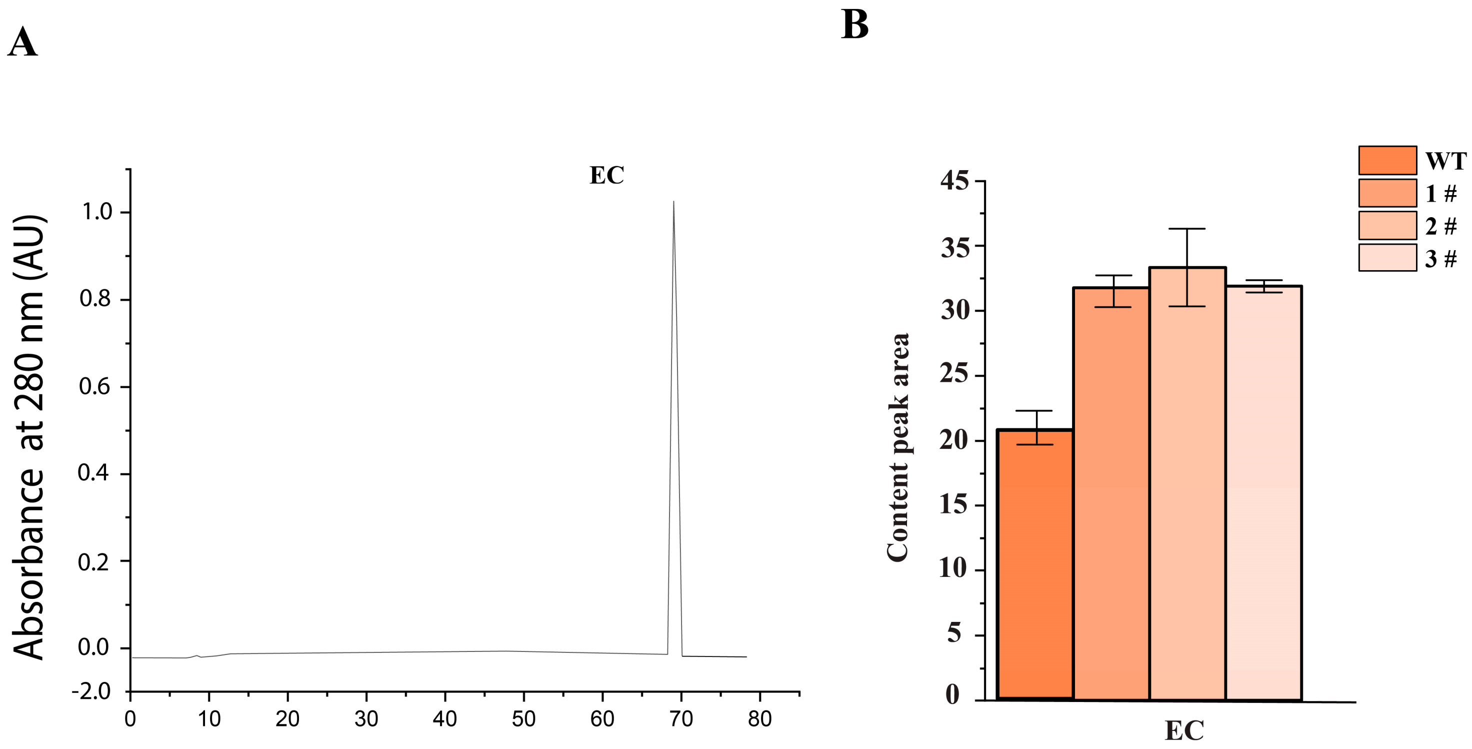

(Supplementary Figure S6): Proanthocyanidin standard and detection in *DkLAC2* transgenic lines and wild-type plants seeds.
